# A phantom study of temperature-dependent MRI T2* measurement

**DOI:** 10.1186/1532-429X-11-S1-P147

**Published:** 2009-01-28

**Authors:** Taigang He, Gillian Smith, John-Paul Carpenter, Raad Mohiaddin, Dudley Pennell, David Firmin

**Affiliations:** CMR Unit, Royal Brompton Hospital and Imperial College London, London, UK

**Keywords:** Phase Array Coil, Thalassemia Patient, Myocardial Iron, Cardiac Iron, Exponential Decay Curve

## Introduction

The measurement of cardiac iron is essential for preventing disease and managing iron-chelating treatment in thalassemia patients. A T2* technique has been developed and clinically validated for this purpose [1, 2]. This method has demonstrated reproducibility and accuracy [2–4] and been increasingly used in clinical practice. However, this technique has not been calibrated against myocardial biopsy because of the risk, the heterogeneity of myocardial iron distribution, and sampling errors. Although autopsy study in hearts donated after death or after cardiac transplantation in thalassemia patients can be performed for calibration, there remain concerns of post-mortem changes in the myocardium and extrinsic variations. Another major issue is the effect of temperature difference from in-vivo to ex-vivo. In a recent post mortem study [5], the *ex-vivo* T2* value was shown to be significantly higher than that matched *in-vivo* measurement. However, the *ex-vivo* measurement was performed at a room temperature of 25°C. Since temperature can potentially affect the calibration, there is the need to investigate this issue. To date we are unaware of any reports regarding myocardium T2* measurements at different temperatures. The purpose of this study, therefore, was to use a carefully designed phantom to determine this relationship.

## Methods

This study was carried out on a 1.5 T whole-body Siemens Sonata system equipped with high performance gradients having a maximum strength of 40 mT/m and maximum slew rate of 200 T/m/s on each axis independently. A four-element cardiac phased array coil was used.

**Figure 1:**
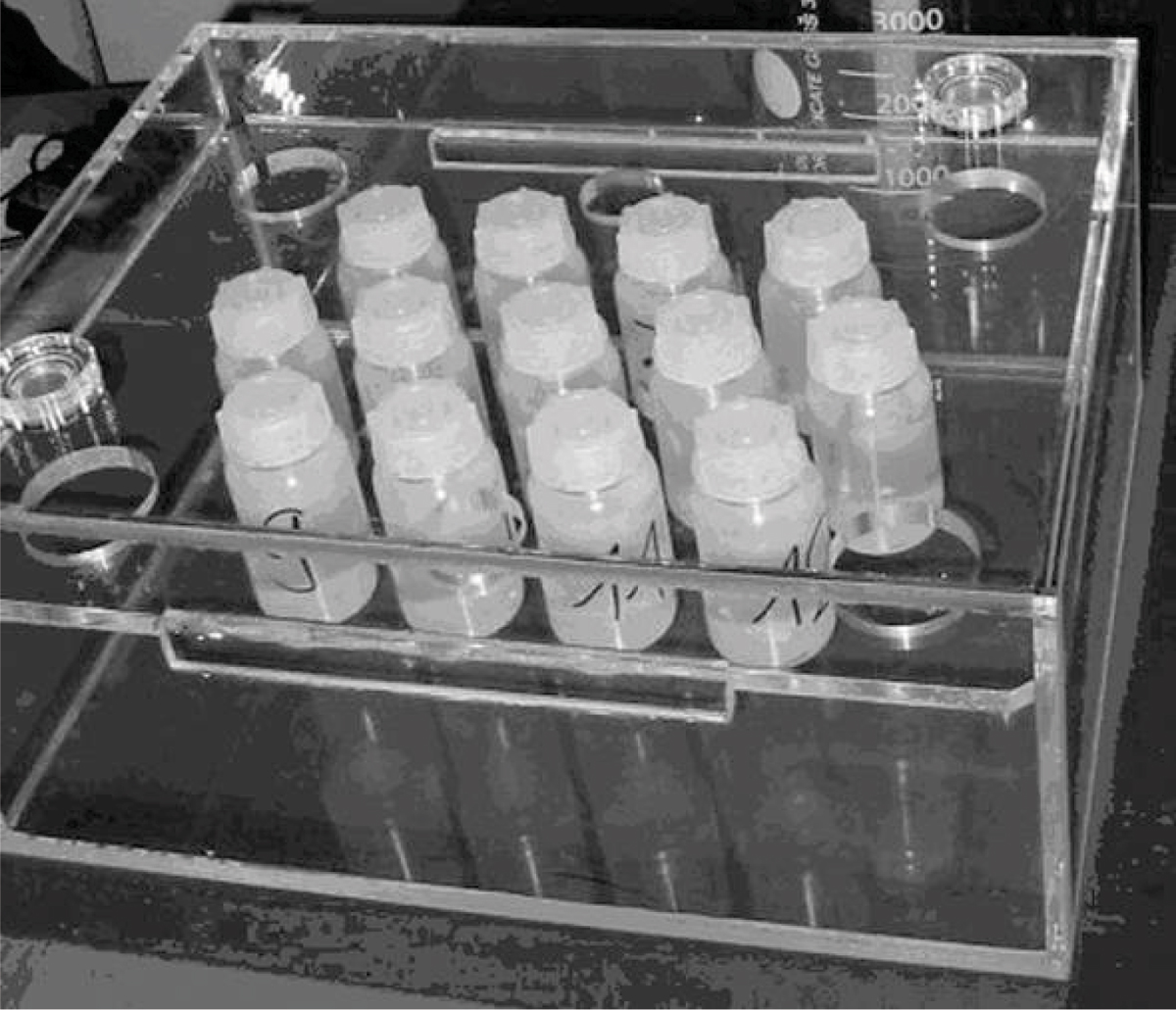
**Photograph of the phantom**.

**Figure 2:**
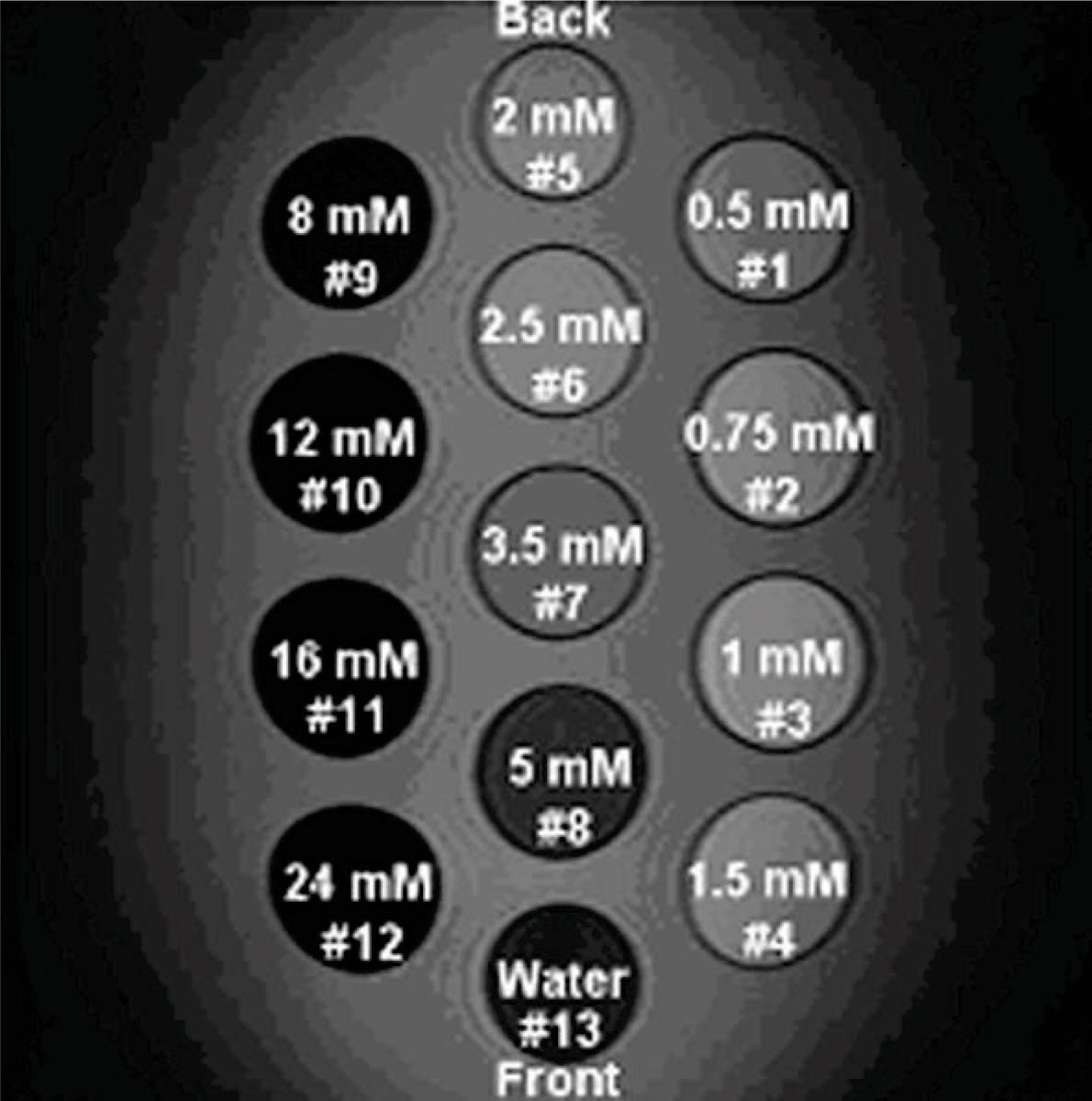
**MnCl2 concentration**.

The phantom consists of a Plexiglas container holding 13 bottles (Figure 1) with various concentrations of MnCl_2_ arranged as depicted in Figure 2. The MnCl_2_ concentrations range from 0 to 24 mM according to the scheme in Figure 2. All solutions contain 0.03% NaN_3_ (sodium acid) in order to prevent growth of bacteria or fungi.

The phantom was immersed in warmed water to reach the temperature of 38°C and then taken out of the water for the scan. The phantom was scanned using the T2* sequence and a coronal image was positioned near the middle of the bottle. During the scan, the phantom was monitored by a temperature probe with an accuracy of 0.1°C continuously until the phantom cooled down to a temperature of 23.0°C. Eight bottles with MnCl_2_ concentrations from 0 to 5 mM were selected for analysis in this study. For T2* measurement, a region of interest was chosen in the vicinity of the center for each bottle. The mean signal intensity of ROI was measured in the series of increasing TE images, and the data were plotted against the TEs to form an exponential decay curve. Nonlinear curve fitting was employed to obtain all T2* measurements of the phantom.

## Results

**Figure 3:**
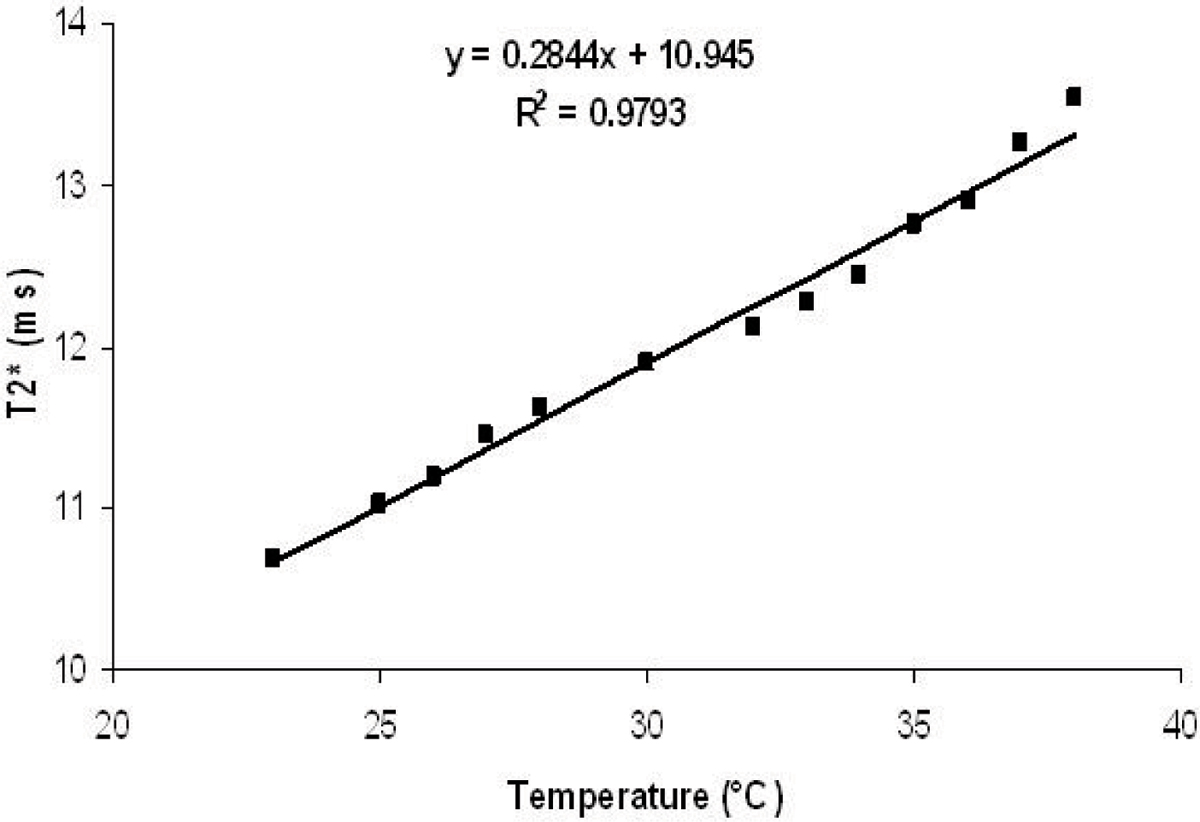
The temperature dependence of T2* measurements in the phantom.

There is clear temperature dependence of T2* measurements. Figure 3 demonstrates that T2* increases linearly with temperature. From 23.0°C to 38.0°C, the maximum T2* change is 23%, which is approximately 1.5% increase per degree Celsius.

## Conclusion

This study has demonstrated that T2* measurement is highly dependent on temperature. In post mortem studies, therefore, the temperature should be set the same as body temperature to avoid significant errors. Further studies are necessary to investigate this relationship in human myocardium.
